# Telomere-to-telomere and haplotype-resolved genome assembly of the Chinese cork oak (*Quercus variabilis*)

**DOI:** 10.3389/fpls.2023.1290913

**Published:** 2023-11-02

**Authors:** Longxin Wang, Lei-Lei Li, Li Chen, Ren-Gang Zhang, Shi-Wei Zhao, Han Yan, Jie Gao, Xue Chen, Yu-Jun Si, Zhe Chen, Haibo Liu, Xiao-Man Xie, Wei Zhao, Biao Han, Xiaochun Qin, Kai-Hua Jia

**Affiliations:** ^1^ School of Biological Science and Technology, University of Jinan, Jinan, China; ^2^ Key Laboratory of Crop Genetic Improvement & Ecology and Physiology, Institute of Crop Germplasm Resources, Shandong Academy of Agricultural Sciences, Jinan, China; ^3^ Shandong Saienfu Stem Cell Engineering Group Co., Ltd, Jinan, China; ^4^ Yunnan Key Laboratory for Integrative Conservation of Plant Species with Extremely Small Populations/Key Laboratory for Plant Diversity and Biogeography of East Asia, Kunming Institute of Botany, Chinese Academy of Sciences, Kunming, Yunnan, China; ^5^ Department of Plant Physiology, Umeå Plant Science Centre, Umeå University, Umeå, Sweden; ^6^ The Second Affiliated Hospital of Shandong First Medical University, Taian, China; ^7^ Chinese Academy of Sciences (CAS), Key Laboratory of Tropical Forest Ecology, Xishuangbanna Tropical Botanical Garden, Chinese Academy of Sciences, Menglun, China; ^8^ Weifang Academy of Agricultural Sciences, Weifang, China; ^9^ InvoGenomics Biotechnology Co., Ltd., Jinan, China; ^10^ Jinan Academy of Landscape and Forestry Science, Jinan, China; ^11^ Key Laboratory of State Forestry and Grassland Administration Conservation and Utilization of Warm Temperate Zone Forest and Grass Germplasm Resources, Shandong Provincial Center of Forest and Grass Germplasm Resources, Jinan, China; ^12^ Department of Ecology and Environmental Science, Umeå Plant Science Centre, Umeå University, Umeå, Sweden

**Keywords:** *Quercus variabilis*, genome assembly, telomere-to-telomere, gap-less, haplotype-resolved

## Abstract

The *Quercus variabilis*, a deciduous broadleaved tree species, holds significant ecological and economical value. While a chromosome-level genome for this species has been made available, it remains riddled with unanchored sequences and gaps. In this study, we present a nearly complete comprehensive telomere-to-telomere (T2T) and haplotype-resolved reference genome for *Q. variabilis*. This was achieved through the integration of ONT ultra-long reads, PacBio HiFi long reads, and Hi-C data. The resultant two haplotype genomes measure 789 Mb and 768 Mb in length, with a contig N50 of 65 Mb and 56 Mb, and were anchored to 12 allelic chromosomes. Within this T2T haplotype-resolved assembly, we predicted 36,830 and 36,370 protein-coding genes, with 95.9% and 96.0% functional annotation for each haplotype genome. The availability of the T2T and haplotype-resolved reference genome lays a solid foundation, not only for illustrating genome structure and functional genomics studies but also to inform and facilitate genetic breeding and improvement of cultivated *Quercus* species.

## Introduction

The genus *Quercus* (Fagaceae) comprises at least 435 species, many of which are forest trees or shrubs, making it one of the most diverse plant genera in the Northern Hemisphere (Denk et al., 2017). The distribution of *Quercus* (Oaks) includes continents such as Asia, North America, Europe, and Africa (Nixon, 2006). Because of its wide range and the high diversity of species, *Quercus* is considered to be an example of successful evolutionary adaptation (Denk et al., 2017; Kremer and Hipp, 2020). *Quercus* is a keystone genus and plays crucial roles in the structure and functioning of forest ecosystems. Additionally, oaks provide many important economic resources, such as timber, tannins, edible nuts, medicinal plants, and bark.

*Q. variabilis*, commonly known as Chinese cork oak, is a fast-growing, drought-tolerant tree species that thrives in harsh soil conditions (Zhou et al., 2010). It has a wide distribution range in China, spanning from Liaoning in the north to Guangdong and Yunnan in the south. This species plays a significant role in warm-temperate deciduous broadleaf forests and subtropical evergreen-deciduous broadleaf mixed forests, making important contributions to maintaining ecosystem stability and protecting biodiversity (Fujiwara and Harada, 2015). In addition to its ecological value, *Q. variabilis* holds economic importance. The thick cork bark of Chinese cork oak can be peeled and used to produce cork stoppers, which are widely utilized in the wine industry (Pereira, 2011). Furthermore, Chinese cork oak is highly valued as a timber species. Its wood is sought after for various applications, including furniture, flooring, and construction. *Q. variabilis*, with its adaptability to harsh environments, ecological significance, and economic value, holds immense potential for ecological restoration, biodiversity conservation, and sustainable resource utilization. The integration of advanced genomic techniques and traditional breeding approaches would pave the way for maximizing the benefits derived from this remarkable tree species.

Previously, we employed PacBio High-Fidelity (HiFi) and high-throughput chromosome conformation capture (Hi-C) data to assemble a chromosome-level genome of *Q. variabilis* (Han et al., 2022). However, there are still several hundred un-anchored contigs in the assembled genome, which have the potential to impact the investigations of the molecular function and evolution of the species. Moreover, despite the chromosomal-level assembly, the high degree of heterozygosity (estimated to be 2.15% (Han et al., 2022)) inherent in the assembly could potentially result in the generation of certain chimeric features during the assembly of a single genome. In this study, we employed a combination of sequencing data including Hi-C technology reads, PacBio HiFi long reads, and Oxford Nanopore Technologies (ONT) ultra-long (N50~100 kb) reads to reassemble the genome of *Q. variabilis*. We generated a telomere-to-telomere (T2T), gap-less, and haplotype-resolved *Q. variabilis* genome. The two haplotypes were the 788.78 Mb and 768.47 Mb in length, with contig N50 length of 64.88 Mb and 55.55 Mb, respectively. 36,830 and 36,370 protein-coding genes were predicted, of which 95.9% and 96.0% were functionally annotated. Our updated assembly, *Q. variabilis* v2.0, represents the first T2T, haplotype-resolved, and gap-less reference genome in *Quercus* and shows higher levels of completeness and contiguity than the previous released *Quercus* genomes. The availability of the nearly complete genome resource of *Q. variabilis* presented in this study will have direct implications for future functional investigations and provide valuable insights for molecular breeding and enhancement of cultivated resources.

## Results

### Assembly of a T2T and haplotype-resolved genome, and architecture of centromeres and telomere regions

**Figure 1:**
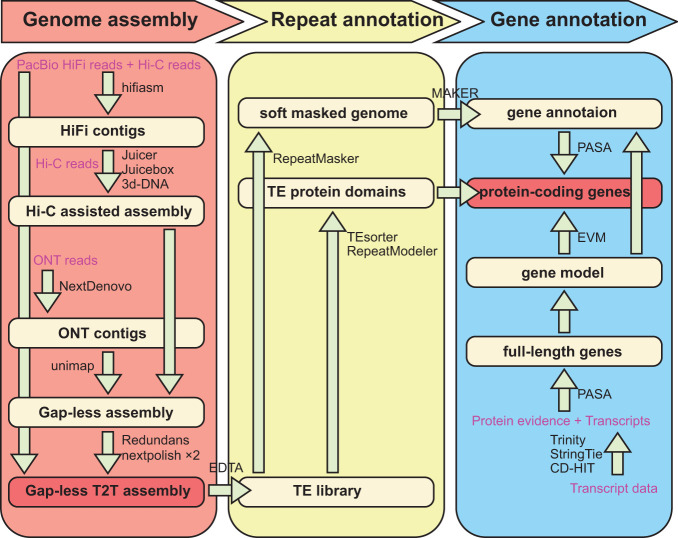
Workflow of genome assembly and annotation.

In this study, we generated 14Gb (17.5X) of ultra-long ONT reads with an average length and N50 of 100kb using the PromethION platform. In addition to the these ultra-long reads, we leveraged 64Gb (~80X) of HiFi long reads, 117Gb (~150X) of Hi-C paired-end reads, and 127Gb (~160X) of DNBSEQ short reads from previously published studies (Han et al., 2022). We initially employed the *de novo* assembler Hifiasm (Cheng et al., 2021) to generate contigs from the PacBio HiFi reads and Hi-C reads (**Figure 1**). Subsequently, we utilized Juicer (Durand et al., 2016) to align the Hi-C reads to the contig generated in the previous step, followed by preliminary Hi-C assisted chromosome assembly using 3d-DNA (Dudchenko et al., 2017). To ensure accuracy, we manually inspected and adjusted the alignment in Juicebox (Robinson et al., 2018). We then utilized 3d-DNA once again to reconstruct the scaffolds, and through manual adjustment in Juicebox, carefully placed the sequences in the correct orientation and position (Robinson et al., 2018).

**Figure 2:**
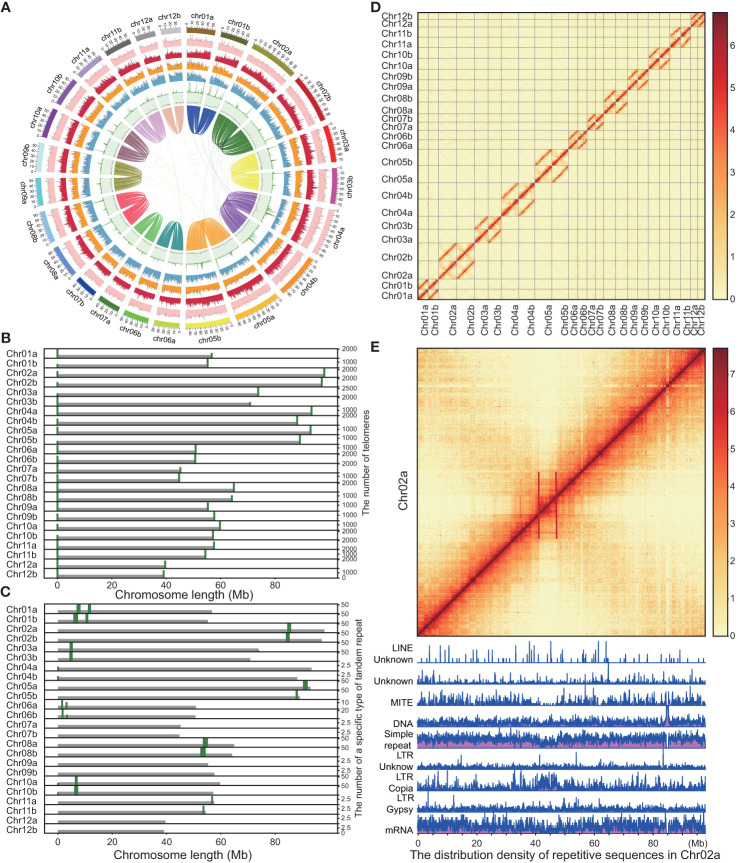
Landscape of genome features of the *Quercus variabilis* genome. **(A)** The genome circle plot displays the circular representation of a genome with the outermost ring representing the chromosome scale. The outer layer of colored blocks is a circular representation of the 12 homologous chromosomes, with thick mark labeling each 10 Mb. From outside to inside: TE density, Class I TE density, Class II TE density, density of protein-coding genes, density of tandem repeats, GC content and collinear blocks (> 100kb). Windows of 500 kb were used for the computation of all statistics. **(B)** Distribution and number of telomeres on *Q. variabilis* chromosomes at 20 kb resolution. **(C)** Distribution and number of a specific type of tandem repeat at 20 kb resolution. **(D)** Heatmap showing Hi-C interactions at 100 kb resolution. **(E)** Heatmap and karyotype map of chromosome 2a at 100 kb and 50 kb resolution, respectively. The strong interaction signal observed in the anti-diagonal signifies common interactions between the arms of the chromosome. This is an indication of the Rabl configuration that interphase chromosomes in this organism have adopted. This phenomenon is commonly attributed to the mechanical tension exerted by the centromeres during metaphase, thereby suggesting that the chromosomal regions demarcated by the red lines likely correspond to the locations of the centromeres.

We employed NextDenovo (https://github.com/Nextomics/NextDenovo) to correct the long ONT reads, and subsequently used the NextDenovo to assemble these reads into contigs. While our assembly successfully yielded nearly complete chromosomes, we encountered challenges due to the repetitive nature of telomere sequences. To overcome this, we reassembled the HiFi reads aligned in the vicinity of the telomeres, leading to extensions in their length. Additionally, we utilized GetOrganelle (Jin et al., 2020) to assemble complete chloroplast and mitochondrial genomes. Eventually, we generated a haplotype-resolved, gap-less, and T2T-level genome of *Q. variabilis* (v2.0). This assembly comprises two haplotypes, each consisting of 12 chromosomes, with a total length of 1.56 Gb (**Figure 2A**; **Table 1**). We attempted to differentiate the two haplotype genomes by using SubPhaser (Jia et al., 2022) to identify subgenome-specific repetitive sequences. However, our findings revealed a lack of subgenome-specific repetitive sequences between the two haplotype genomes. Consequently, we arbitrarily designated the allelic chromosomes as haplotype genome A and B. The contig N50 of the haplotype A and B genomes (65 and 56 Mb) in *Q. variabilis* v2.0 were twice that of v1.0 (26 Mb), while the genome length of each haplotype remained similar (789 Mb versus 768 Mb). Remarkably, our haplotype-resolved reference genome assembly had only nine gaps (three and six gaps, respectively). Through the search for the canonical ‘TTTAGGG/CCCTAAA’ telomeric repeat, we identified 48 distinct telomeres, with CCCTAAA at the 5’ end and TTTAGGG at the 3’ end (**Figure 2B**). A total of 64 inversions, 1,600 translocations, and 1,530 duplications were detected between two haplotype genomes, encompassing regions of 15.11, 15.30, and 9.43 Mb. Furthermore, we identified a total of 5.1 million single nucleotide polymorphisms (SNPs) variation.

**Table 1:** Genome assembly statistics of *Quercus variabilis*.

Genome feature	Haplotype A	Haplotype B
Genome size (bp)	788,783,505	768,469,721
Chromosome number	12	12
Contig number	17	18
Contig N50 (bp)	64,884,478	55,550,940
Contig L50	5	6
Contig N90 (bp)	39623673	39,048,944
Contig L90	11	12
Scaffold number	14	12
Scaffold N50 (bp)	64,884,478	64,144,329
Scaffold L50	5	5
Scaffold N90 (bp)	45,195,692	44,771,246
Scaffold L90	11	11
Gap number	3	6
Gene number	36,830	36,370
LAI	21.83	21.88

In the realm of three-dimensional genomic architecture, the physical bending or folding of chromosomes can bring gene regions, which are physically distant, into closer spatial proximity. This phenomenon is vividly represented as strong interaction signals on Hi-C heat maps (Pal et al., 2019; Oluwadare et al., 2020). Centromeres, a crucial part of the chromosomes, often induce interaction signals at greater distances on Hi-C heat maps. This effect is attributed to their roles in pairing and pulling during cell division (Varoquaux et al., 2015). Furthermore, plant centromeres are typically composed of tandem repeat monomers that exhibit high frequencies of chromosomal interactions (Melters et al., 2013). In our study, we identified a highly repetitive tandem sequence of 367 characters, which manifests a relatively high frequency across different chromosomes, using the Tandem Repeats Finder tool v4.09 (Benson, 1999) (**Figure 2C**). However, we could not match the position of these repetitive sequences with the interaction of chromosomes on the Hi-C map (**Figure 2D**). For instance, we saw a stronger interaction signal near the middle of chromosome 2A (**Figures 2D, E**), but the highly tandem repeat unit appears near the end of chromosome 2A (**Figure 2C**). In addition, we utilized karyoploteR (Gel and Serra, 2017) to construct a karyotype chart for identifying whether a specific repeat dominates the centromere sequence (Repeat annotation can be found in the following text), but our results showed that the particular repeat did not appear in the stronger chromosomal interaction signal region (**Figure 2E** is an example of this). Thus, the repeat sequence may not be the primary determinant for the characteristics of the centromere sequence. It should be noted that karyotyping and analysis of chromosomal interaction signal regions are just some of the methods used to determine whether a specific repeat dominates the centromere sequence, and additional techniques may be necessary to further investigate this phenomenon.

### Genome annotation

Our analysis revealed 316.56 Mb and 313.43 Mb of repeat sequences, which accounted for 54.24% and 53.86% of the *Q. variabilis* haplotype A and B genome, respectively (**Table 1**). These proportions are consistent with previously published annotations (Han et al., 2022). In these repetitive sequences, long terminal repeat retrotransposons (LTR-RTs) constitute the highest proportion, accounting for approximately 30% of the genomic sequences. Haplotypes A and B contain 313,570 and 303,993 LTR-RTs, respectively, with total lengths reaching 243,439,472 and 234,877,351. Meanwhile, Gypsy and Copia collectively represent 23% of the genome size (**Table 2**). The repetitive sequence annotation can be obtained from https://doi.org/10.6084/m9.figshare.22313569.v2.

**Table 2:** Genome repeats statistics of *Quercus variabilis*.

Type	Order	Superfamily	Number	Length (bp)	Percent (%)	Mean length (bp)
Haplotype A	LTR	Copia	94530	82439742	10.46	872.1
		Gypsy	96667	100996928	12.81	1044.79
		Retrovirus	3	242	0	80.67
		unknown	122370	60002560	7.61	490.34
	LINE	LINE	1096	518718	0.07	473.28
	DNA	Helitron	216661	59517634	7.55	274.7
	TIR	EnSpm CACTA	42705	14519242	1.84	339.99
		MuDR Mutator	94990	29908792	3.79	314.86
		PIF Harbinger	38972	13423293	1.7	344.43
		Tc1 Mariner	4541	1326274	0.17	292.07
		hAT	50491	18880896	2.4	373.95
	Unknown	Unknown	70075	27001080	3.43	385.32
	Simple repeat	Simple repeat	453499	15841286	2.01	34.93
	Low complexity	Low complexity	63893	3140081	0.4	49.15
	total		1350493	427516768	54.24	316.56
Haplotype B	LTR	Copia	90767	78753106	10.25	867.64
		Gypsy	95006	98094095	12.76	1032.5
		Retrovirus	2	155	0	77.5
		unknown	118218	58029995	7.55	490.87
	LINE	LINE	1068	481994	0.06	451.31
	DNA	Helitron	211796	57860397	7.53	273.19
	TIR	EnSpm CACTA	41631	14127907	1.84	339.36
		MuDR Mutator	92556	29108683	3.79	314.5
		PIF Harbinger	38252	13100453	1.7	342.48
		Tc1 Mariner	4414	1323406	0.17	299.82
		hAT	49439	18391909	2.39	372.01
	Unknown	Unknown	68277	25987510	3.38	380.62
	Simple repeat	Simple repeat	446219	15521845	2.02	34.79
	Low complexity	Low complexity	62835	3099540	0.4	49.33
	total		1320480	413880995	53.86	313.43

After masking the repeated sequences, a total of 36,830 and 36,370 protein-coding genes were predicted in haplotype A and B genomes, respectively (**Table 1**). The protein-coding genes had an average coding sequence length of 5,102 bp and an average of 4.5 exons per gene. We predicted non-coding RNA sequences, which resulted in the prediction of 641 ribosomal RNA (rRNA), 1,282 transfer RNA (tRNA) and 1,764 other non-coding RNA (ncRNA).

By integrating the functional annotations from three methods, we successfully annotated 95.90% and 96.00% of the protein-coding genes on haplotypes A and B, respectively, leaving 4.1% and 4.0% of the protein-coding genes unannotated for their functions. These gene function annotations will provide a valuable resource for breeders and researchers, who can query the data and analyze their gene of interest through https://doi.org/10.6084/m9.figshare.22313569.v2.

### Assessment of haplotype-resolved genome completeness

**Figure 3:**
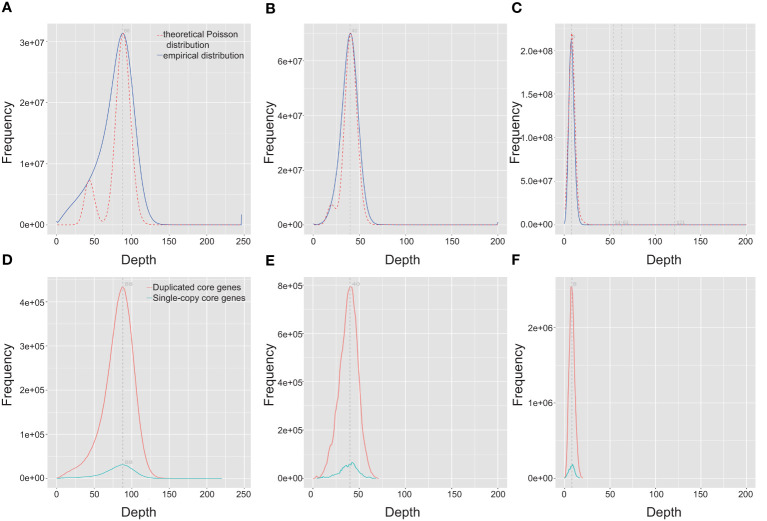
Distribution of the coverage depth of the whole *Quercus variabilis* genome. Distribution of coverage depth by mapping DNBSEQ, HiFi, and ONT data onto the *Q. variabilis* genome **(A–C)** and the BUSCO core gene region **(D–F)**.

To evaluate the completeness of *Q. variabilis* v2.0 genome assembly, we initially employed BUSCO v5.3.2 (Simão et al., 2015) based on the embryophyta_odb10 lineage dataset to assess the completeness of two haplotype genomes. Out of the 1,614 expected genes from the embryophyta_odb10 lineage, the proportions of complete BUSCOs were 98.4% and 98.3% for the two haplotype genomes, respectively (**Table 3**). The protein-coding genes were also evaluated using BUSCO. The proportion of complete core genes in the two haplotype genes was 97.4% and 97.9%, respectively. Additionally, we calculated the BUSCO values based on the eudicots_odb10 lineage and obtained similar results (**Table 3**). By utilizing the LTR assembly index (LAI) v2.9.0 (Ou et al., 2018) score, we evaluated our haplotype genome assembly and found that the scores for both haplotypes were 21.83 and 21.88, respectively, reaching the “gold standard” (**Table 1**). Subsequently, we utilized BWA v0.7.17-r1188 (Li, 2013), and Minimap2 (Li, 2018) to align short, and long/ultra-long reads to the genome. The results indicated that over 99.69% of the reads can be mapped to the genome, covering more than 99.91% of the genomic regions (**Table 4**). The coverage depths of all loci conformed to a Poisson distribution and there was no obvious heterozygous peak (**Figures 3A–C**). The coverage depth of both single-copy and multi-copy BUSCO genes is consistent and does not show any significant heterozygous peaks (**Figures 3D–F**). These indicate that our assembly does not exhibit redundancy or collapse. We also used Juicer (Durand et al., 2016) to map Hi-C paired-end reads back to the genome and confirmed that no macroscopic assembly errors were present (**Figure 2D**).

**Table 3:** The BUSCO evaluation statistics of two haplotypes and their protein-coding genes using two distinct core datasets, namely embryophyta and eudicots.

	embryophyta lineage				eudicots lineage			
Type	Haplotype A genome	Haplotype B genome	Haplotype A protein-coding genes	Haplotype B protein-coding genes	Haplotype A genome	Haplotype B genome	Haplotype A protein-coding genes	Haplotype B protein-coding genes
Complete BUSCOs	1588 (98.4%)	1587 (98.3%)	1572 (97.4%)	1580 (97.9%)	2290 (98.4%)	2285 (98.2%)	2255 (96.9%)	2252 (96.8%)
Complete and single-copy BUSCOs	1527 (94.6%)	1527 (94.6%)	1046 (64.8%)	1111 (68.8%)	2182 (93.8%)	2185 (93.9%)	1536 (66.0%)	1577 (67.8%)
Complete and duplicated BUSCOs	61 (3.8%)	60 (3.7%)	526 (32.6%)	469 (29.1%)	108 (4.6%)	100 (4.3%)	719 (30.9%)	675 (29.0%)
Fragmented BUSCOs	14 (0.9%)	16 (1.0%)	14 (0.9%)	9 (0.6%)	14 (0.6%)	17 (0.7%)	16 (0.7%)	18 (0.8%)
Missing BUSCOs	12 (0.7%)	11 (0.7%)	28 (1.7%)	25 (1.5%)	22 (1.0%)	24 (1.1%)	55 (2.4%)	56 (2.4%)
Total BUSCO groups searched	1614 (100%)	1614 (100%)	1614 (100%)	1614 (100%)	2326 (100%)	2326 (100%)	2326 (100%)	2326 (100%)

**Table 4:** Map rate and coverage statistics of different types of sequencing reads.

Data set	reads mapped (%)	properly paired (%)	bases mapped (%)	genome coverage ≥ 1× (%)	genome coverage ≥5× (%)	genome coverage ≥10× (%)	genome coverage ≥20× (%)
HiFi	99.74%	–	99.69%	99.97%	99.93%	99.79%	97.57%
ONT	99.85%	–	99.86%	99.91%	90.32%	31.22%	0.10%
DNBSEQ	99.97%	99.72%	99.97%	99.96%	99.81%	99.40%	97.79%

**Figure 4:**
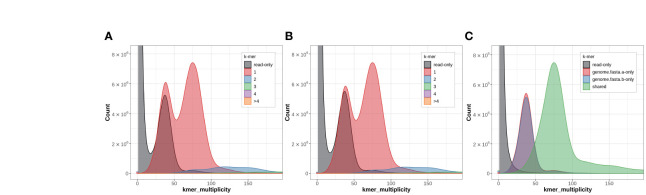
Genome quality assessment via the Merqury spectral plot. A copy number spectral plot for the haplotype A **(A)** and B **(B)** assemblies of *Quercus variabilis*. **(C)** An assembly spectral plot for the evaluation the completeness of *k*-mers.

To further ensure the quality of the genome assembly, we conducted Merqury (Rhie et al., 2020) analysis (using *k* = 19) based on long reads. The consensus quality values (QVs) for the individual haplotype A, B and AB were 50.96, 52.10, and 51.48, respectively (**Figure 4**). Additionally, the *k*-mer completeness scores for the individual genomes A, B and AB were 79.35, 78.19, and 98.52, respectively.

These findings demonstrate the high quality, completeness, and reliability of the *Q. variabilis* genome assembly.

## Discussion

In this study, we reported the telomere-to-telomere and haplotype-resolved genome assembly of the Chinese cork oak. This comprehensive genomic resource will have direct implications for future functional investigations as well as molecular breeding efforts of cultivated *Quercus* species.

Firstly, by integrating various novel sequencing technologies including ONT ultra-long reads, PacBio HiFi long reads, and Hi-C chromosome conformation data, we have successfully constructed a high-quality T2T and haplotype-resolved genome assembly of Chinese cork oak. This provides a paradigm for plant genome assembly approaches.

Secondly, we have comprehensively annotated both haplotype genomes, including repetitive sequences, non-coding RNAs, and protein-coding genes. Both genomes showed functional gene annotation rates exceeding 95%. These annotations provide the foundation for downstream analyses.

Thirdly, we evaluated the completeness and contiguity of the genome assembly using multiple methods. The results demonstrated that both haplotype genome assemblies have reached “gold standard” quality. The high-quality genome paves the way for subsequent investigations into gene family evolution, genomic structural variations, and functional genomics.

Fourthly, our study revealed abundant structural variations between the two haplotype genomes. This is likely associated with the high heterozygosity of Chinese cork oak. Further elucidation of these variations will facilitate understanding the formation mechanisms of heterosis in plants.

In summary, the high-quality Chinese cork oak genome obtained in this study provides a valuable resource for tree genomics research. We look forward to multi-disciplinary and multi-level follow-up studies based on this genome, to propel molecular breeding efforts of Chinese cork oak.

## Materials and methods

### Sample collection, library preparation, and sequencing

Leaf tissue samples were collected from a wild tree that was over 400 years old, located in the Culaishan National Forest Park, Shandong Province, China. The leaf was immediately frozen in liquid nitrogen and stored at -80°C until further processing. Genomic DNA was extracted using a modified CTAB method as described in a previous study (Doyle and Doyle, 1987). Library preparation was carried out using an Oxford Nanopore SQK-LSK109 kit, and ultra-long reads were generated on the PromethION platform.

### *De novo* genome assembly

We performed genome assembly using a combination of various software tools (**Figure 1**). The PacBio HiFi long reads and Hi-C short reads were utilized as a combined input for the genome assembler Hifiasm v0.16.1-r375 (Cheng et al., 2021). The assembly process was performed Hi-C mode with default parameters, resulting in a pair of haplotype-resolved assembly contigs. The two assembled haplotype contig files were merged and further processed by mapping Hi-C reads to them using Juicer v1.6 (Durand et al., 2016), and a preliminary Hi-C-assisted chromosome assembly was generated using 3d-DNA v180922 (Dudchenko et al., 2017) with parameters “–early-exit -m haploid -r 0”. The accuracy of the chromosome segmentation boundaries and assembly errors were manually checked and adjusted using Juicebox v1.11.08 (Robinson et al., 2018).

To further enhance the quality of the assembly, the NextDenovo (https://github.com/Nextomics/NextDenovo) was utilized for correcting ultra-long ONT reads and assembling them into contigs. These contigs were then mapped back to the genome sequence using the unimap (https://github.com/lh3/unimap) software to bridge any remaining gaps in the assembly. Then ultra-long ONT reads were used to close remained gaps with LR_Gapcloser (Xu et al., 2019). Due to incomplete or missing telomere assemblies in some cases, we utilized Hifiasm to reassemble contigs from HiFi reads near the chromosome telomeres and aligned them back to the scaffolds to extend the telomere sequence as much as possible. In addition, we assembled the complete mitochondrial (420 kb) and chloroplast (164 kb) genomes using the GetOrganelle software (Jin et al., 2020), in conjunction with DNBSEQ and HiFi data. At this point, we have preliminarily assembled the nuclear, mitochondrial, and chloroplast genomes. Subsequently, we mapped the DNBSEQ short reads back to the assembly and employed NextPolish v1.3.1 software (Hu et al., 2020) to perform two rounds of genome polishing. Afterward, we used Redundans v0.13c (Pryszcz and Gabaldon, 2016) software to remove redundancy.

### Assessment of genome completeness

To evaluate the completeness of *Q. variabilis* v2.0 genome assembly, we initially employed BUSCO v5.3.2 (Simão et al., 2015) based on the embryophyta_odb10 and eudicots_odb10 lineage dataset, LTR Assembly Index (LAI) (Ou et al., 2018), Merqury (using *k* = 19) analysis (Rhie et al., 2020) to assess the completeness of two haplotype genomes. We also utilized BWA v0.7.17-r1188 (Li, 2013), and Minimap2 (Li, 2018) to align short, and long/ultra-long reads to the genome, aiming to assess the integrity of the genome by analyzing the mapping rate and coverage depths.

### Repeat annotation

We employed the *de novo* transposable element (TE) annotation pipeline EDTA v1.9.9 (Ou et al., 2019) (–sensitive 1 –anno 1), which integrates homology-based and structure-based approaches, to identify families of TEs and generate a TE library. We then utilized RepeatMasker v4.0.7 (Chen, 2004) to further identify TEs and other repeat sequences with the library and the default parameters.

### Genes prediction and functional annotation

For RNA sequencing, a total of 168 Gb RNA sequencing data was obtained from the NCBI SRA database (SRR13123715-SRR13123726, SRR15860171-SRR15860176, and SRR19749981), as well as from previous sequencing experiments (Han et al., 2022). To construct a comprehensive transcriptome of *Q. variabilis*, we used two distinct methods. Firstly, we employed *de novo* transcriptome assembly using Trinity v2.0.6 (Grabherr et al., 2011). Secondly, we performed assembly guided by the *Q. variabilis* reference genome, using StringTie v1.3.5 (Pertea et al., 2015). The two distinct methods yielded 402,099 and 146,900 transcripts, respectively. We obtained 461,163 non-redundant transcriptomes using CD-HIT v4.6 (Li and Godzik, 2006) with 95% identity and 95% coverage.

We employed a combined strategy to predict protein-coding genes in this study, using three distinct lines of evidence: transcriptome, protein homology, and *ab initio* prediction. We downloaded the protein homology of *Q. lobata* (Sork et al., 2016), *Q. robur* (Plomion et al., 2018), *Q. suber* (Ramos et al., 2018), *Q. mongolica* (Ai et al., 2022), *Q. acutissima* (Fu et al., 2022; Liu et al., 2022), *Q. variabilis* (Han et al., 2022), *Castanea mollissima* (Wang et al., 2020), *Juglans regia* (Zhang et al., 2022b), *Betula pendula* (Salojärvi et al., 2017), *Morella rubra* (Jia et al., 2019), *Arabidopsis thaliana* (Cheng et al., 2017), and *Vitis vinifera* (Jaillon et al., 2007), and generated a total of 276,330 non-redundant protein sequences using CD-HIT with 95% identity and 95% coverage.

Based on the transcript evidence, the gene structure was annotated using the PASA v2.4.1 process (Haas et al., 2003). Full-length genes were identified by comparing them with the above proteins (at least 95% coverage). The full-length gene set was used to train the parametric model of AUGUSTUS v3.4.0 (Stanke et al., 2008) with five rounds of optimization.

The MAKER v2.31.9 pipeline (Cantarel et al., 2008) was utilized for gene annotation firstly, incorporating *ab initio* prediction, transcript evidence, and homologous protein evidence. Briefly, the genome was masked using RepeatMasker (Chen, 2004) with the above library from EDTA, and AUGUSTUS (Stanke et al., 2008) was employed for *ab initio* prediction of protein-coding genes. Transcripts obtained above was aligned to the repeat-masked genome using BLASTN, while protein sequences were aligned using BLASTX (Altschul et al., 1990). The resulting annotations were further refined using Exonerate v2.2.0 (Slater and Birney, 2005), and hints files were generated based on these results. With the evidence-based hints, the final predicted gene models was generated using AUGUSTUS (Stanke et al., 2008) as implemented in the MAKER pipeline.

Considering the relatively low accuracy of the MAKER pipeline, we further integrated MAKER (Cantarel et al., 2008) and PASA (Haas et al., 2003) annotations using EVidenceModeler (EVM) v1.1.1 (Haas et al., 2008) to generate a consistent gene annotation. To avoid the introduction of TE coding regions, we used TEsorter (Zhang et al., 2022a) to identify TE protein domains on the genome and mask them with EVM. Untranslated region (UTR) and alternative splicing were added to the EVM (Haas et al., 2008) annotation using PASA (Haas et al., 2003). Genes were removed if they containing internal stop codons, lacked start codons, lacked stop codons, or those < 50 amino acids in length. Annotation of the tRNA, rRNA and other various non-coding RNAs was performed using tRNAScan-SE v1.3.1 (Lowe and Eddy, 1997), barrnap (https://github.com/tseemann/barrnap), and RfamScan (Lowe and Eddy, 1997), respectively.

To annotate the protein-coding gene functions, we employed three strategies: mapping the protein-coding genes to the eggNOG v5.0 orthologous gene database using eggNOG-mapper v2.0.1 (Cantalapiedra et al., 2021), mapping protein-coding genes to various protein databases (including Swiss_Prot, TrEMBL, NR and the Arabidopsis TAIR10 database) using diamond v0.9.24 (Buchfink et al., 2021) (identity > 30%, E-value < 1e-5), and mapping protein-coding genes to the InterPro subdatabase (PRINTS, Pfam, SMART, PANTHER and CDD) using InterProScan v5.27-66.0 (Jones et al., 2014).

### Genome comparison between haplotype assemblies

We used the minimap2 v2.24-r1122 (Li, 2018) algorithm to perform alignments between haplotype assemblies, while SyRI v1.4 (Goel et al., 2019) was employed for detecting syntenic regions and structural variation. The structural rearrangements identified were visualized using Plotsr v0.5.4 (Goel and Schneeberger, 2022).

## Data availability statement

The raw sequence data reported in this paper have been deposited in the Genome Sequence Archive (Chen et al., 2021b) in National Genomics Data Center (Members and Partners, 2022), China National Center for Bioinformation / Beijing Institute of Genomics, Chinese Academy of Sciences (GSA: CRA009472) that are publicly accessible at https://ngdc.cncb.ac.cn/gsa and NCBI BioProject PRJNA990843. The haplotype-resolved genome sequence data reported in this paper have been deposited in the Genome Warehouse in National Genomics Data Center (Chen et al., 2021a; Members and Partners, 2022), Beijing Institute of Genomics, Chinese Academy of Sciences / China National Center for Bioinformation, under accession number GWHBQTN00000000 that is publicly accessible at https://ngdc.cncb.ac.cn/gwh. Split haplotype genome assemblies and annotation can also be obtained from NCBI BioProject PRJNA990841 and PRJNA990842. The gene function annotation and repetitive sequence annotation are accessible at https://doi.org/10.6084/m9.figshare.22313569.v2.

## Author contributions

LW: Writing – original draft, Software, Writing – review & editing. L-LL: Data curation. LC: Data curation. R-GZ: Software. S-WZ: Software. HY: Data curation. JG: Software. XC: Data curation. Y-JS: Data curation. ZC: Software. HL: Writing – review & editing. X-MX: Conceptualization. WZ: Software. BH: Conceptualization. XQ: Writing – review & editing. K-HJ: Writing – original draft, Writing – review & editing.
